# 7.R. Health promotion interventions

**DOI:** 10.1093/eurpub/ckac130.133

**Published:** 2022-10-25

## Abstract

This abstract has been withdrawn

